# Interactions in cancer treatment considering cancer therapy, concomitant medications, food, herbal medicine and other supplements

**DOI:** 10.1007/s00432-021-03625-3

**Published:** 2021-04-17

**Authors:** Clemens P. J. G. Wolf, Tobias Rachow, Thomas Ernst, Andreas Hochhaus, Bijan Zomorodbakhsch, Susan Foller, Matthias Rengsberger, Michael Hartmann, Jutta Hübner

**Affiliations:** 1 Klinik für Innere Medizin II, Hämatologie und Internistische Onkologie, Universitätsklinikum Jena, Am Klinikum 1, 07747 Jena, Germany; 2 Klinik für Innere Medizin II, Hämatologie und Internistische Onkologie, Pneumologie, Universitätsklinikum Jena, Am Klinikum 1, 07747 Jena, Germany; 3 Klinik für Innere Medizin II, Hämatologie und Internistische Onkologie, Konservative Tagesklinik des UniversitätsTumorCentrums (UTC), Universitätsklinikum Jena, Am Klinikum 1, 07747 Jena, Germany; 4 Onkologische Kooperation Harz, Kösliner Straße 14, 38642 Goslar, Germany; 5 Klinik für Urologie, Universitätsklinikum Jena, Am Klinikum 1, 07747 Jena, Germany; 6 Klinik und Poliklinik für Frauenheilkunde und Fortpflanzungsmedizin, Universitätsklinikum Jena, Am Klinikum 1, 07747 Jena, Germany; 7 Apotheke des Universitätsklinikums, Universitätsklinikum Jena, Am Klinikum 1, 07747 Jena, Germany; 8 Klinik für Innere Medizin II, Hämatologie und Internistische Onkologie, Integrative Onkologie, Universitätsklinikum Jena, Am Klinikum 1, 07747 Jena, Germany

**Keywords:** Drug–drug interactions, Complementary and alternative medicine, Food–drug interactions, Cancer treatment, Chemotherapy, Cancer outpatients

## Abstract

**Purpose:**

The aim of our study was to analyse the frequency and severity of different types of potential interactions in oncological outpatients’ therapy. Therefore, medications, food and substances in terms of complementary and alternative medicine (CAM) like dietary supplements, herbs and other processed ingredients were considered.

**Methods:**

We obtained data from questionnaires and from analysing the patient records of 115 cancer outpatients treated at a German university hospital. Drug–drug interactions were identified using a drug interaction checking software. Potential CAM-drug interactions and food–drug interactions were identified based on literature research.

**Results:**

92.2% of all patients were at risk of one or more interaction of any kind and 61.7% of at least one major drug–drug interaction. On average, physicians prescribed 10.4 drugs to each patient and 6.9 interactions were found, 2.5 of which were classified as major. The most prevalent types of drug–drug interactions were a combination of QT prolonging drugs (32.3%) and drugs with a potential for myelotoxicity (13.4%) or hepatotoxicity (10.1%). In 37.2% of all patients using CAM supplements the likelihood of interactions with medications was rated as likely. Food-drug interactions were likely in 28.7% of all patients.

**Conclusion:**

The high amount of interactions could not be found in literature so far. We recommend running interaction checks when prescribing any new drug and capturing CAM supplements in medication lists too. If not advised explicitly in another way drugs should be taken separately from meals and by using nonmineralized water to minimize the risk for food–drug interactions.

## Introduction

Improvements in cancer treatment lead to decreasing disease-specific mortality rates for many types of cancer. The overall increasing life expectancy results in more cancer diagnoses in old age (Robert Koch-Institut and Gesellschaft der epidemiologischen Krebsregister in Deutschland e.V. 2019). Both often require polypharmacy for an extensive treatment of concomitant diseases and a treatment of side effects of conventional anticancer drugs. The World Health Organization stated that more than 50% of all patients do not take their medications in a correct manner and that more than 50% of all medicines worldwide are inappropriately prescribed, sold or dispensed. Reasons are for example the usage of too many drugs per patient, a mismatch between clinical guidelines and prescribed medications or an inadequate self-medication by the patients (World Health Organization 2006, pp. 16–17). A meta-analysis of 39 studies showed that side effects in drug therapy are the fourth to sixth most common cause of death in the USA (World Health Organization 2006, p. 10). Beside the issues with polypharmacy especially in elderly patients, the use of complementary and alternative medicine (CAM) is associated with younger age (Molassiotis et al. 2005; Wode et al. 2019). A European study revealed that 36% of cancer patients used CAM (Molassiotis et al. 2005). In several studies the most common applied CAM modalities included the intake of ingredients like herbs or supplements or included a special dietary (Alsanad et al. 2016; Naing et al. 2011; Zeller et al. 2013). According to a study by Zeller et al. (2013), 65% of the patients taking CAM supplements were at risk of CAM–drug interactions (“likely” and “possible”) related to their cancer therapy. This did not include other medications, e.g. for comorbidities.

The likelihood or severity of interactions of CAM supplements with conventional medications is discussed controversially for many CAM supplements due to inconsistent data or a lack of clinical studies.

Literature data found only referred to a small partial aspect of all possible causes of interactions. Several studies either examined drug–drug interactions, drug–food interactions or interactions between CAM supplements and conventional therapy. No data were found combining all of these aspects in cancer patients while analysing the likelihood or severity of interactions.

The aim of our study was to analyse the frequency and the severity of potential interactions in outpatient cancer therapy and to elaborate the most frequently observed combinations of drugs which may unintentionally interact. Therefore, we assessed several sources of interactions in cancer patients’ therapy to our investigation: the conventional cancer treatment prescribed by physicians, concomitant medications, food and CAM including dietary supplements, herbs and other processed ingredients. As a result we expected a higher number of patients affected by interactions of any kind than reported in literature. The relevance might be underestimated so far.

## Methods

### Patients

A random sample of 115 oncological outpatients with different cancer diagnoses who received therapy in March or April 2020 at the University Hospital of Jena, Germany, were included in this cross-sectional study. The patients were informed and agreed to participate in this investigation. They were interviewed by a standardized questionnaire and agreed in examining their patient records.

### Data collection

Demographic data, the type of cancer diagnosis, the time since initial diagnosis, the use of complementary and alternative medicine (CAM), the temporal relationship between food and drug intake, the consumed liquids for drug intake and the current medications prescribed by physicians were recorded in the questionnaire. Regarding CAM only the use of CAM substances was asked which might cause interactions with other drugs. Other CAM modalities such as acupuncture, prayer or massage were not captured. In addition, patients’ records were analysed detecting further information about the diagnosis and the current medications especially anticancer medications administered.

### Evaluation of interactions

Patients’ medication lists were screened referring to interactions using “i:fox” version 3.33.10.1239 (2020) provided by “ifap Service-Institut für Ärzte und Apotheker GmbH” Martinsried/Munich, Germany. The severity of interactions within the medical treatment was classified according to “i:fox” program: minor (1); moderate (2); major (3). CAM substances were analysed based on extensive literature research for each drug with all active ingredients. The probability of interactions involving CAM was classified either unlikely (0), or possible (1), or likely (2). In the case that the probability of a particular interaction was assessed heterogeneously in the literature, two of the authors (Wolf and Huebner) discussed and decided on a classification. Therefore, the conditions of the respective studies and the reasons given by the authors were taken into account. Consensus was reached in every case. Regarding food–drug interactions only those interactions were included and discussed which were rated as likely considering the patients’ information from the questionnaire according to literature data.

All interactions registered were differentiated into three major categories:

- 1. Interactions within the medical treatment (drug–drug interactions)
  - 1.1 Interactions between cancer treatment and concomitant diseases’ treatment
  - 1.2 Interactions within the treatment of concomitant diseases
- 2. Interactions between CAM supplements and medical treatment (CAM–drug interactions)
  - 2.1 Interactions between CAM supplements and cancer treatment
  - 2.2 Interactions between CAM supplements and concomitant diseases’ treatment
- 3. Interactions between food and the medical treatment (food–drug interactions)

All drug–drug interactions (category 1) and interacting drug combinations were listed and differentiated regarding their frequency and their severity. Category 2 and 3 interactions were classified according to the probability of their existence.

Interactions within drugs of the cancer treatment such as an additive potential for QT prolongation, myelotoxicity or hepatotoxicity were interpreted as tolerated and known risks and were only analysed if there was an additional risk in combination with concomitant medications. Desired additive drug effects reported as interactions by the drug interaction checking software within the medical therapy were excluded like additive blood pressure lowering in patients with antihypertensive treatment. Medications in context with receiving cancer treatment such as premedication were not included in our study under the assumption that these drugs are applied only temporarily and following a standardized protocol in which potential interactions with cancer treatment are known and accepted. On-demand medication was included in the analysis even as patients might use it less frequently. Drugs prescribed for treatment of cancer side effects or anticancer drugs’ side effects such as nausea were analysed within the category of drugs for concomitant diseases’ treatment. All active ingredients of the drugs or therapeutic regimes prescribed in the medical treatment by physicians were considered separately for interaction checks. If a prescribed drug contained several active ingredients, these were counted as individual drugs.

### Statistics

Three datasets were completed using Microsoft Office Excel 2016 for listing the results: one with all patients’ variables, one with all drug interactions found and one for analysing CAM supplements. Statistical analyses were performed using IBM SPSS Statistics 27. Associations were assessed using Cramér’s V (φc) after running Fisher’s Exact Test or the Fisher-Freeman-Halton Test for categorical or ordinal variables, Spearman’s correlation coefficient (rs) for numerical variables and Eta Squared (*η*^2^) in case of associations between numerical and categorical or ordinal variables completed by performing an analysis of the variance. A statistical model using binary logistic regression was assessed for analysing CAM–drug interactions.

In a second article we evaluate and report the data of the patients with respect to possible predictors of CAM supplements’ usage and potential interactions of CAM supplements with cancer treatment.

## Results

One hundred fifteen patients participated in our investigation. Demographic data are shown in Table 1. The mean age was 61 years (SD = 13.3). The most prevalent cancer diagnoses were breast cancer (*n* = 25), other gynecological cancers (*n* = 15) such as ovarian cancer, endometrial cancer or cervical cancer, multiple myeloma (*n* = 15) and leukemia (*n* = 10).

**Table 1:** Demographic data (*n* = 115)

Age	Years
Median	63
Range	18–86
Gender	*n* (%)
Male	47 (40.9%)
Female	68 (59.1%)
Marital status	*n* (%)
Single	11 (9.6%)
Firm relationship	8 (7.0%)
Married	76 (66.1%)
Divorced	6 (5.2%)
Widowed	13 (11.3%)
No data	1 (0.9%)
School leaving qualification	*n* (%)
No degree	1 (0.9%)
After 8th grade (Hauptschulabschluss)	10 (8.7%)
After 10th grade (Mittlere Reife)	41 (35.7%)
After 12th or 13th grade (Abitur)	31 (27.0%)
No data	32 (27.8%)
Type of cancer diagnosis	*n* (%)
Breast cancer	25 (21.7%)
Other gynecological cancer	15 (13.0%)
Multiple Myeloma	15 (13.0%)
Leukemia	10 (8.7%)
Pancreatic cancer	8 (7.0%)
Gastrointestinal cancer	8 (7.0%)
Renal cancer	8 (7.0%)
Cholangiocellular carcinoma	6 (5.2%)
Lung cancer	6 (5.2%)
Malignant lymphoma	5 (4.3%)
Others	9 (7.8%)

**Fig. 1:**
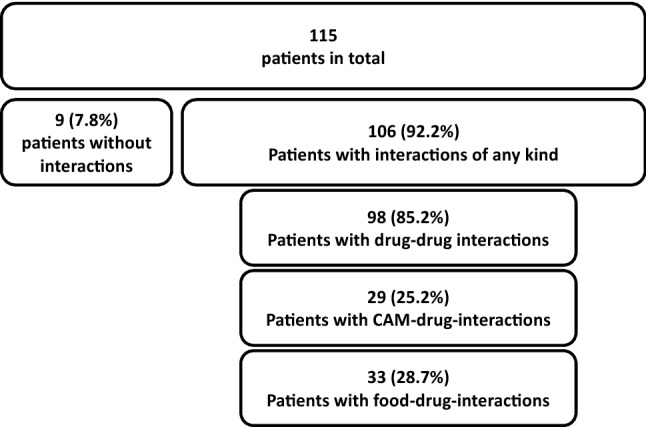
Overview on the prevalence of potential interactions

In total, 106 of all 115 patients had at least one interaction of any kind affecting the medical treatment of the patient (Fig. 1). This equals 92.2% of all patients.

### Category 1: Interactions within the medical treatment

One hundred fifteen patients took 1191 active pharmaceutical ingredients prescribed by physicians, which equals 10.4 in average per patient (SD = 4.4, range 1–23, median 10). These ingredients can be divided into 279 (23.4%) agents prescribed for cancer therapy (average per patient 2.4, SD = 1.3, range 0–5) and 912 (76.6%) ingredients prescribed for the treatment of concomitant diseases or for the treatment of side effects of the cancer therapy (average per patient 7.9, SD = 3.9, range 0–19).

In 98 of the 115 patients (85.2%), drug interactions were found with one or more major interaction in 71 patients (out of 115 in total, 61.7%).

In total, 799 interactions were identified with the interaction checking software (Table 2) for 445 different drug combinations, excluding interactions within substances for cancer treatment. Zero to 44 interactions were found per outpatient with an average amount of almost 7 interactions (6.9, SD = 7.9). 35.9% (287 out of 799) of all interactions were classified as major in severity with 2.5 major interactions per patient in average (SD = 3.6, range 0–17).

**Table 2:** Frequency of interacting drug combinations within the medical treatment of 115 patients

***1 Interactions within the medical treatment***					
In total	*n* = 799		6.9 in average per patient	SD = 7.9	Range 0–44
Major interactions	*n* = 287	35.9%	2.5 in average per patient	SD = 3.6	Range 0–17
***1.1 Interactions between cancer treatment and concomitant diseases' treatment***					
In total	*n* = 251		2.2 in average per patient	SD = 3.0	Range 0–16
Major interactions	*n* = 74	29.5%	0.6 in average per patient	SD = 1.7	Range 0–10
***1.2 Interactions within the treatment of concomitant diseases***					
In total	*n* = 548		4.8 in average per patient	SD = 6.0	Range 0–36
Major interactions	*n* = 213	38.9%	1.9 in average per patient	SD = 2.8	Range 0–11

Two hundred fifty-one interactions (out of 799 in total) were detected between cancer treatment and concomitant diseases’ treatment with 74 (out of 251, 29.5%) classified as major interactions.

Five hundred forty-eight interactions (out of 799 in total) were identified within the treatment of concomitant diseases with 213 (38.9%) of them classified as major. Within concomitant diseases’ treatment drugs up to 36 interacting drug combinations could be seen in one patients’ medication list.

**Fig. 2:**
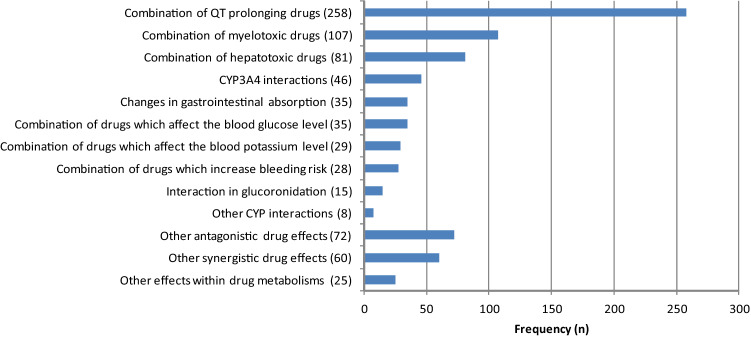
Types of interacting drug combinations (*n* = 799) within the medical treatment

The prevalence of the different types of interactions is shown in Fig. 2. The most frequent types of drug–drug interactions were a combination of QT prolonging drugs (32.3%) and a combination of drugs with a potential for myelotoxicity (13.4%) or hepatotoxicity (10.1%).

Approximately one-third (258 out of 799, 32.3%) of all interactions identified were due to a combination of drugs which potentially cause QT prolongation—not including those within different cancer treatment drugs. In total, 232 drugs with a potential for QT prolongation were prescribed—in average about 2 per patient (SD = 1.6, range 0–6). 10.3% of them (24 out of 232) were substances for cancer treatment.

One hundred seven combinations of substances with a potential for myelotoxicity were discovered in 227 prescribed drugs with a potential for myelotoxicity. That equals 2.0 potentially myelotoxic drugs per patient in average (SD = 1.3, range 0–6). One hundred forty-one (out of 227, 63.5%) of these were drugs prescribed for cancer treatment.

Eighty-one drug combinations with a potential for hepatotoxicity were identified within 155 drugs prescribed which may act hepatotoxic. Fifty-six of these 155 drugs (36.1%) were prescribed for cancer treatment. The other 99 drugs (63.9%) were taken for concomitant diseases’ treatment.

5.8% (46 out of 799) of all interactions were caused by a combination of cytochrome (CYP) 3A4 acting drugs.

The most prevalent involved drugs in drug–drug interactions were ciprofloxacin, fluconazole, ondansetron and pantoprazole. All of these drugs have a potential for QT prolongation. Ciprofloxacin and fluconazole additionally might act hepatotoxic and are inhibitors of CYP3A4. Pantoprazole and ondansetron are substrates of CYP3A4, while pantoprazole is metabolized by CYP2C19 as well. Fluconazole acts as a CYP2C19 inhibitor.

Table 3 shows all drugs involved in interactions according to the medication reviews for the four most prevalent types of either pharmacodynamic or pharmacokinetic interactions.

**Table 3:** Drugs involved in interacting drug combinations (in alphabetic order)

***Combination of drugs with a potential for QT prolongation (258 combinations among all patients)***	
Cancer therapy	Arsenic trioxide, bendamustine, eribulin, fluorouracil, oxaliplatin
Other therapy	**Sympathomimetics**: indacaterol, formoterol, salmeterol **Psychiatric medications**: amitriptyline, doxepin, escitalopram, melperone, mirtazapine, opipramol, quetiapine, venlafaxine **Proton pump inhibitors**: pantoprazole, omeprazole, esomeprazole **Anti-infectives**: amphotericin B, ciprofloxacin, co-trimoxazole, fluconazole, posaconazole, voriconazole **5-HT3 receptor antagonists**: granisetron, ondansetron **Others**: alfuzosin, pregabalin, tramadol, ebastine, ivabradine
***Combination of drugs with myelotoxicity (107 combinations among all patients)***	
Cancer therapy	**Antibodies**: rituximab, trastuzumab **Anthracyclines**: daunorubicin, doxorubicin, epirubicin **Platin-based**: cisplatin, carboplatin **Pyrimidine analogues**: capecitabine, cytarabine, fluorouracil, gemcitabine **Taxanes**: docetaxel, paclitaxel **Vinca alkaloids**: vinblastine, vincristine **Others**: bleomycin, cyclophosphamide, dacarbazine, etoposide, fludarabine, hydroxycarbamide, irinotecan, methotrexate, mitoxantrone
Other therapy	Acetaminophen, allopurinol, co-trimoxazole, enalapril, glimepiride, hydrochlorothiazide, mesalazine, metamizole, mirtazapine, zoledronic acid, ramipril
***Combination of drugs with hepatotoxicity (81 combinations among all patients)***	
Cancer therapy	**Anthracyclines**: daunorubicin, doxorubicin, epirubicin **Others**: cytarabine, gemcitabine, methotrexate, paclitaxel, topotecan, tretinoin
Other therapy	**Statins**: atorvastatin, simvastatin **Anti-infectives**: ciprofloxacin, co-trimoxazole, metronidazole, fluconazole, voriconazole **ACE inhibitors**: enalapril, lisinopril, perindopril, ramipril **Others**: acetaminophen, lorazepam, testosterone, thiamazole
***CYP3A4 interactions (46 combinations among all patients) (Anticancer pharmaceuticals are written in italics)***	
Substrates	Amlodipine, atorvastatin, clopidogrel, *cyclophosphamide, dexamethasone,* esomeprazole, fentanyl, *irinotecan*, omeprazole, ondansetron, pantoprazole, quetiapine, simvastatin, tramadol, *venetoclax, vinblastine, vincristine*
Inhibitors	Aprepitant, ciprofloxacin, fluconazole, *idelalisib*, ranolazine, voriconazole
Inducers	*Glucocorticoids*

Significant (*p* < 0.050) associations with a medium effect size (0.300 < *φc* < 0.500) (Wei et al. 2019) were found between the type of cancer diagnosis, classified as shown in Table 1, and the presence of drug–drug interactions (*p* = 0.029, *φc* = 0.441) or the presence of major drug–drug interactions (*p* = 0.013, *φc* = 0.429). Some cancer diagnoses were associated with a higher likelihood for patients being affected by major drug–drug interactions as expected: pancreatic cancer (7 patients affected, 4.9 expected, equals 142.9% in comparison to the expected number) and other gynecological cancer than breast cancer (13 affected, 9.3 expected, equals 139.8% in comparison to the expected number). Renal cancer was associated with a lower prevalence of major drug–drug interactions (0 affected, 4.9 expected, 0%). For all other diagnoses' categories, either the difference between the actually affected and the expected number of patients was less than 1 or the difference was less than 10%. In case of pancreatic cancer the patients received either FOLFIRINOX or variations of this regime (6 out of 8) or gemcitabine and paclitaxel (2 out of 8). All patients with gynecological cancer diagnosis other than breast cancer received either carboplatin- or doxorubicin-based cancer treatment. Five out of eight patients with renal cancer received nivolumab only for cancer treatment while the other three were on axitinib and pemprolizumab.

Significant associations were identified between the type of cancer diagnosis and the amount of medications in total (*p* < 0.001, *η*^2^ = 0.281), the amount of anticancer drugs (*p* = 0.001, *η*^2^ = 0.234) or the amount of drugs prescribed for concomitant diseases’ treatment (*p* = 0.001, *η*^2^ = 0.235). This also applies on the statistical relationships between the type of diagnosis and the amount of interactions from category 1.1 (*p* < 0.001, *η*^2^ = 0.279), the amount of major interactions from category 1.1 (*p* < 0.001, *η*^2^ = 0.406), the amount of interactions from category 1.2 (*p* = 0.034, *η*^2^ = 0.165), the amount of major interactions from category 1.2 (*p* = 0.024, *η*^2^ = 0.174), the amount of drug–drug interactions in general (*p* = 0.007, *η*^2^ = 0.200) or the amount of major drug–drug interactions (*p* = 0.013, *η*^2^ = 0.188). Because Eta Squared is greater than 0.140 in all cases, we can assume strong statistical relationships (Wei et al. 2019).

No significant associations were found with regard to the influence of gender on the prevalence of drug–drug interactions (*p* = 0.790, *φc* = 0.047) or on the number of drugs prescribed (*p* = 0.352, *η*^2^ = 0.008).

The number of medications prescribed correlates significantly with the number of interactions (*p* < 0.001, rs = 0.838, CI 95% [0.774,0.883]) and the number of major interactions found (*p* < 0.001, rs = 0.649, CI 95% [0.517,0.749]). The effect size is large in both cases (rs > 0.500) (Wei et al. 2019).

Elderly patients received more medications in total (*p* = 0.052, rs = 0.182, CI 95% [0.003, 0.359]) and significantly more drugs for concomitant diseases’ treatment (*p* = 0.009, rs = 0.242, CI 95% [0.065, 0.410]) but a smaller amount of anticancer drugs (*p* = 0.203, rs = − 0.120, CI 95% [− 0.289, 0.048]). The number of interactions within the treatment of concomitant diseases (*p* = 0.002, rs = 0.293, CI 95% [0.113, 0.467]) and the number of drug–drug interactions in general (*p* = 0.005, rs = 0.260, CI 95% [0.076, 0.428]) were significantly higher in advanced age.

Negative correlations were identified between the time since initial cancer diagnosis and the number of drugs prescribed and the number of interactions found. These correlations involving this period of time revealed being significant regarding the overall number of drugs prescribed per patient (*p* = 0.027, rs = − 0.206, CI 95% [− 0.384, − 0.020]), the number of anticancer drugs (*p* = 0.002, rs = − 0.282, CI 95% [− 0.454, − 0.096]) and regarding the number of interactions between anticancer drugs and drugs prescribed for concomitant diseases' treatment (*p* = 0.002, rs = − 0.282, CI 95% [− 0.440, − 0.094]).

### Category 2: Interactions between CAM supplements and medical treatment

Forty-three of the 115 patients (37.4%) stated to consume additional substances, which were rated as complementary or alternative medicine (CAM).

In 29 of all 43 CAM supplements using patients (67.4%) potential interactions with prescribed medications were discovered (interactions possible or likely). In 16 of the 43 patients (37.2%) the likelihood (likely > possible > unlikely) for interactions with CAM supplements was rated as likely.

A statistical model using binary logistic regression revealed that a higher number of CAM supplements is associated with a higher probability of CAM-drug interaction of any kind (*p* < 0.001, OR = 4.344, CI 95% [2.434, 7.754]) while the number of conventional drugs prescribed by physicians had no influence (*p* = 0.760, OR = 0.978, CI 95% [0.850, 1.126]). The overall model fit was *χ*^2^ = 58.8.

#### 2.1 Interactions between CAM supplements and cancer treatment

In more than half of all CAM using patients (22 out of 43, 51.2%) interactions between cancer therapy and CAM supplements were evaluated. The likelihood was rated as likely in 7 cases as shown in Table 4 and as possible in 15 cases.

**Table 4:** Likely Interactions between CAM supplements and cancer treatment in seven patients

	Anticancer drug	CAM supplement	Interaction (likely)/effect
1	Paclitaxel	Mistletoe	Increased effects of paclitaxel by inhibiting ribosomal protein synthesis (Pae et al. 2001)
2	Bortezomib	Vitamin C	Reduction of effects of bortezomib (Perrone et al. 2009)
	Bortezomib Dexamethasone	Chinese herbs	Chinese herbs are rated as likely interacting, CYP effects likely (Zeller et al. 2013)
	Bortezomib Dexamethasone	Ginger	Increased levels of bortezomib and dexamethasone due to CYP3A4 inhibition by ginger (Cho and Yoon 2015; JANSSEN-CILAG INTERNATIONAL NV 2019; Kimura et al. 2010; Qiu et al. 2015; ratiopharm GmbH 2020a)
3	Bortezomib Dexamethasone	Ginger	Increased levels of bortezomib and dexamethasone due to CYP3A4 inhibition by ginger (Cho and Yoon 2015; JANSSEN-CILAG INTERNATIONAL NV 2019; Kimura et al. 2010; Qiu et al. 2015; ratiopharm GmbH 2020a)
4	Irinotecan	Ginger	Increased levels of irinotecan due to CYP3A4 inhibition by ginger (Cho and Yoon 2015; Kimura et al. 2010; Petri 2017; Qiu et al. 2015)
5 + 6	Doxorubicin	Vitamin C	Reduction of effects of anthracyclines on tumor cells (Heaney et al. 2008; Zeller et al. 2013)
7	Epirubicin	Vitamin C	Reduction of effects of anthracyclines on tumor cells (Zeller et al. 2013)

#### 2.2 Interactions between CAM supplements and concomitant diseases' treatment

Potential interactions between the treatment of concomitant diseases and CAM supplements were documented in 22 patients (22 out of 43, 51.2%). These were rated as likely in 13 patients as shown in Table 5 and as possible in 9 patients.

**Table 5:** Likely interactions between CAM supplements and concomitant diseases' treatment in 13 patients

	Drug	CAM supplement	Interaction (likely)/effect
1	Ciprofloxacin	Magnesium	Reduced absorption of ciprofloxacin (Walker and Wright 1991)
2	CYP acting drugs	Chinese herbs	Chinese herbs are rated as likely interacting, CYP effects likely (Zeller et al. 2013)
	Multiple drugs	Ginger	Several drugs of the patient could interact with ginger^a^
3	Multiple drugs	Ginger	Several drugs of the patient could interact with ginger^a^
4	Ciprofloxacin	Beetroot	Iron, magnesium, calcium: reduced absorption of ciprofloxacin (Walker and Wright 1991)
	Ciprofloxacin	Iron	Reduced absorption of ciprofloxacin (Walker and Wright 1991)
	Multiple drugs	Ginger	Several drugs of the patient could interact with ginger^a^
5	Multiple drugs	Chinese herbs	Chinese herbs are rated as likely interacting, CYP effects likely (Zeller et al. 2013)
	Multiple drugs	Ginger	Several drugs of the patient could interact with ginger^a^
6	Levothyroxine	Minerals	Calcium, iron: reduced absorption of levothyroxine (Benvenga et al. 2017)
	Acetylsalicylic acid	Vitamin E	Increased bleeding risk (Liede et al. 1998)
7	CYP3A4 acting drugs	Nigella sativa	Inhibition of CYP2D6 and CYP3A4 by Nigella sativa (Al-Jenoobi et al. 2010)
8	Multiple drugs	Ginger	Several drugs of the patient could interact with ginger^a^
9	Multiple drugs	Chinese herbs	Chinese herbs are rated as likely interacting, CYP effects likely (Zeller et al. 2013)
10	Ciprofloxacin	Magnesium	Reduced absorption of ciprofloxacin (Walker and Wright 1991)
11	Acetylsalicylic acid	Vitamin E	Increased bleeding risk (Liede et al. 1998)
12	Levothyroxine	Calcium	Reduced absorption of levothyroxine (Benvenga et al. 2017)
13	Multiple drugs	Chinese herbs	Chinese herbs are rated as likely interacting, CYP effects likely (Zeller et al. 2013)

^a^Ginger: increased bleeding risks (e.g. with warfarin, NSAIDs) (Hodges and Kam 2002; Huebner 2012, p. 180; Shalansky et al. 2007); additional hypotonic effects (Huebner 2012, p. 180; Suekawa et al. 1984) with antihypertensive drugs; possibly additional sedating effects with sedating drugs; effects on drug glucoronidation (Huebner 2012, p. 180); CYP interactions regarding CYP2C9, CYP2C19 and CYP3A4 effecting multiple drugs (e.g. amlodipine, atorvastatin, pantoprazole) (Cho and Yoon 2015; Kim et al. 2012; Kimura et al. 2010)

### Category 3: Interactions between food and the medical treatment

28.7% (33 out of 115) of all patients were at risk of at least one food–drug interaction. About half of all patients (58 out of 115, 50.4%) indicated that they take their drugs with their meals. This might be one critical source for food–drug interactions.

Three patients received bortezomib of which one drank green tea for drug intake. The effect of bortezomib might be reduced in combination with green tea and especially green tea extracts (Golden et al. 2009).

Ciprofloxacin was prescribed in 24 patients. Fourteen of these patients (14 out of 58, 24.1%) took their drugs with their meals and 2 others used mineral water. This can reduce the ciprofloxacin absorption due to complex formations with several metal ions (El-Sabawi et al. 2019; Walker and Wright 1991). One patient stated drinking beetroot juice for drug intake which includes interacting cations such as iron, magnesium and calcium (Usman et al. 2015; Walker and Wright 1991). The drug intake regarding food-drug interactions with ciprofloxacin was rated as harmless for seven of all patients using this drug (7 out of 24, 29.2%).

Twenty patients were on levothyroxine. Half of these patients (10 out of 20, 50.0%) indicated taking their drugs with their meals. In four cases they were combined with mineral water intake and in one case with milk. Dairy products and soy products should not be consumed together with levothyroxine (Fruzza et al. 2012; Neuvonen et al. 1991). Certain food can be problematic due to interactions with metal ions like iron or calcium (Benvenga et al. 2017; Skelin et al. 2017) such as for ciprofloxacin with these cations. For one fourth of the 20 patients their levothyroxine intake revealed being harmless (5 out of 20, 25.0%).

Spironolactone was prescribed in five patients. There is an elevated risk for hyperkalemia by using spironolactone (Yang et al. 2018) and especially for taking spironolactone and an ACE inhibitor (Abbas et al. 2015) such as in three of the five patients. Together with food rich in potassium like tomatoes and bananas (Górska-Warsewicz et al. 2019; Herbold and Edelstein 2008, p. 446) or impaired renal function this risk might be even increased (Dixit et al. 2019).

Regarding CYP3A4 interactions no patient stated drinking grapefruit juice but it remains unknown whether there are other sources of food like certain marmalade which might cause CYP3A4 interactions as well (Bailey et al. 2013).

No significant associations were found with regard to food–drug interactions—neither for age, nor for gender, nor for marital status, nor for school leaving qualification, nor for the category of cancer diagnosis.

## Discussion

The high proportion of 92% of all patients with drug interactions of any kind and 62% of all patients with drug–drug interactions classified as major could not be found in literature so far. Hoemme et al. (2019) report a number of 24% of cancer patients affected by severe drug–drug interactions and Castro-Manzanares et al. (2019) report a number of 27% of outpatients affected by at least one clinically relevant drug–drug interaction. The differences to our study might partly result from considering all patients’ medications including on-demand medication and the use of a different drug interaction checking software. The most prevalent drugs involved in drug–drug interactions in our investigation were ciprofloxacin, fluconazole, pantoprazole and ondansetron. Beside pantoprazole these drugs were often prescribed as on-demand medication—in case of infection after permission by a physician or in case of nausea—so they do not represent a constant risk for the patients but should be considered by the physicians regarding interactions as well. Multiple sources for interactions were found while combining these drugs: cytochrome interactions, QT prolongation or a potential for hepatotoxicity concerning ciprofloxacin and fluconazole. In addition to myelotoxicity these types of drugs’ side effects were the ones most prevalent seen.

Schaefer et al. (2018) discovered that 47% of 202 hematological cancer patients had at least one potentially interacting drug combination, which is more than discovered in other studies (e.g. Castro-Manzanares et al. 2019; Hoemme et al. 2019). Two hundred two patients with 1929 drugs were included in the study (Schaefer et al. 2018) while our study includes 115 patients with 1191 active ingredients in medical treatment. That equals 9.5 drugs per patient in the study from Schaefer et al. (2018) versus 10.4 drugs per patient in average in our investigation. We discovered a higher number of drugs prescribed per patient being associated with more drug–drug interactions as it is described in literature as well (Hoemme et al. 2019). This might partly cause a higher rate of patients affected by drug interactions in our study. Another reason might be a difference in mean age of the patients: 61 years in our study compared to 56 in the study from Schaefer et al. (2018). A higher mean age might result in a polypharmaceutical situation with a higher number of drugs prescribed due to a higher number of concomitant diseases in elderly patients according to significant associations found in our study.

A different setting of our study might affect the amount of interactions as well. While Schaefer et al. (2018) analysed patients’ discharge letters after hospitalization, our study was carried out in an outpatient setting, where patients are receiving medications from different specialized physicians and general practitioners. We discovered that there often was an incomplete recording of patients’ medications in the outpatient setting while receiving cancer therapy. The medication lists of the physicians were often not congruent with the medication lists we received from the patients themselves for this study. This might be a source for unaware clinically relevant drug interactions if the physicians do not know all patients’ medications. In addition, outpatients treated at university hospitals in Germany compared to patients treated by an outpatient resident oncologist might require a more extensive treatment of concomitant diseases due to a more complex course of illness or patients’ constitution requiring more medications in treatment.

The interaction checking tool applied was the one which found the highest amount of drug–drug interactions in comparison to three other tools tested via random samples. The utilized tool seems to have a relevant impact on the amount of results and the amount of interactions found in different studies. The drug interaction checking software listed all combinations of drugs with a potential for QT prolongation. A prolongation of QT time might present as dizziness, syncope or even ventricular tachycardia (torsade de pointes) (Altmann et al. 2008). In our opinion this is important to consider for cancer treatment especially when prescribing anticancer drugs with a potential for cardiotoxicity. A QT prolongation caused by interacting concomitant medications might be misinterpreted as a side effect of anticancer medications leading to dose reduction of cancer therapy or even interrupting the administration of the regime. In one patient from our study a QT prolongation was seen as clinically relevant and a change in concomitant medications by the oncologists was able to reduce QT time so the patient could receive arsenic trioxide for his leukemia treatment further on. About one-third (32%) of all interactions identified were a combination of QT prolonging drugs.

For some drugs the likelihood of interactions is controversial between literature data and drug information by the manufacturers. For example, for the proton pump inhibitor pantoprazole the manufacturers state the likelihood for agranulocytosis between 1:1000 and 1:10,000 (e.g. AbZ-Pharma GmbH 2020; ratiopharm GmbH 2020b) while only some case reports could be found showing this side effect for pantoprazole (Gouraud et al. 2010) and esomeprazole (Yu et al. 2018) although these drugs are broadly used. The drug interaction checking tool did not identify this as a risk as well as a retrospective analysis (Ozkan et al. 2015). In comparison, the risk of agranulocytosis of metamizole is stated at less than 1:10,000 by the manufacturers (e.g. AbZ-Pharma GmbH 2019; HEUMANN PHARMA GmbH & Co. Generica KG 2019). For proton pump inhibitors such as pantoprazole there are also ambiguous data about the potential for QT prolongation. While the interaction checking software claimed this as a potential risk of pantoprazole in combination with other QT prolonging drugs as well as the German “Arzneimittelbrief” (2016) the manufacturers do not mention that as a risk in the drug information for physicians (e.g. AbZ-Pharma GmbH 2020; ratiopharm GmbH 2020b).

Significant associations discovered between the type of cancer and the amount of interactions or the amount of drugs prescribed might partly result from the different therapeutic regimes these patients received for cancer therapy. Antibodies like nivolumab and pembrolizumab used in patients with renal cancer are less toxic and have a lower potential for interactions than drugs of the FOLFIRINOX regime used in patients with pancreatic cancer, or carboplatin- or doxorubicin-based cancer therapy used in patients with gynecological cancer entities other than breast cancer. As a result, more drugs might be required in the treatment of side effects of anticancer drugs, which can interact as well. The attending physician might play an additional role: During our study we noticed that certain physicians tended to always prescribe the same medications as an accompanying medication for cancer therapy, which then led to interactions in several patients with the same type of cancer. Drugs most frequently involved in interactions such as ciprofloxacin, fluconazole, pantoprazole and ondansetron were prescribed by the physicians who also administered the cancer therapy. These physicians should, therefore, pay special attention to possible interactions and carefully check the indications.

The significantly higher amount of medications and especially of medications prescribed for concomitant diseases’ treatment most probably results from an increased number of comorbidities in higher age. In contrast, elderly patients received a lower number of anticancer drugs, which might be caused by an increased number of comorbidities as well. Certain organ dysfunctions may require omission or dose reduction in cytotoxic cancer therapy. Negative correlations between the time since initial cancer diagnosis and the number of drugs prescribed or the number of interactions found might be explained by a less aggressive type of cancer these patients suffered from which leads to an increased survival time while requiring less medications.

There are very little and partly heterogeneous data in the literature for many CAM substances, even for commonly used CAM substances like vitamin supplements. The clinical relevance and prevalence of interactions often remains unknown due to missing clinical data while only theoretical concepts exist for example based on murine models or in vitro studies. Even if there is a risk discovered by in vivo studies it was often not mentioned in the official drug information for physicians in Germany. For example, the manufacturer of Velcade® (bortezomib) is not reporting the potential risk of reduced effects of bortezomib while taking vitamin C (Perrone et al. 2009) or green tea extracts (Golden et al. 2009) in the information of bortezomib for physicians (JANSSEN-CILAG INTERNATIONAL NV 2019).

A study on gynecological cancer outpatients in Germany which used a similar classification system and reviewed similar CAM supplements as we did, revealed about two-thirds (65%) of all patients using CAM supplements (“likely” and “possible”) being at risk of interactions between CAM and anticancer drugs (Zeller et al. 2013). We discovered 51% of all patients using CAM supplements having at least one potential CAM-drug interaction involving cancer treatment and 67% being at risk of one or more CAM–drug interaction of any kind. Because of the small number of patients using CAM in our study (43 out of 115 patients) random effects might influence our results but the high number of potential interactions including CAM supplements either with cancer treatment or with concomitant medications exemplifies the relevance of assessing CAM supplements in routine cancer care. The prevalence of potential CAM–drug interactions was influenced by the number of CAM supplements taken but not by the number of drugs prescribed by physicians. It is important to ask patients about their CAM use even they receive only a small amount of conventional medication to identify CAM-drug interactions.

Food–drug interactions were likely in 29% of all patients. The patients mostly were at risk for interactions in case of drugs combined with food rich in polyvalent cations in case of ciprofloxacin and levothyroxine. Different cations can reduce the absorption of these drugs by complexation such as aluminum, iron or calcium (Benvenga et al. 2017; El-Sabawi et al. 2019; Skelin et al. 2017; Walker and Wright 1991). Interactions might be clinically less relevant when the dosage prescribed is based on serum levels while consistently taking the drugs in the same manner. For example, the combination of levothyroxine with milk might be harmless when always drinking milk for drug intake and adapting the dosage based on resulting serum medication level by physicians. This type of interactions can be prevented if patients would take ciprofloxacin and levothyroxine at time intervals away from a meal and with nonmineralized water. The manufacturer's recommendations on such time intervals must be followed regarding any drug. Regarding the potential interaction of green tea with bortezomib a reduced or even neutralized anticancer effect of boronic acid-based proteasome inhibitors was discovered while taking polyphenols such as EGCG which are constituents of green tea (Golden et al. 2009). Although there are doubts that brewed green tea causes sufficiently high serum levels of polyphenols to negatively affect bortezomib (Shah et al. 2009) we want to mention it as a possible risk especially for green tea extracts just like it is described in information for patients (e.g. LHRM e.V. 2014). In addition, green tea extracts can act hepatotoxic (García-Cortés et al. 2016; Mazzanti et al. 2009) and cause cytochrome interactions as well (Chung et al. 2009; Engdal and Nilsen 2009; Shin and Choi 2009).

The estimation of the World Health Organization that more than 50% of all medications are prescribed, sold or dispensed inappropriately (World Health Organization 2006, pp. 16–17) is highlighted by our results to be plausible regardless a currently unidentified dark number of unaware interactions.

### Limitations

Although we tried to consider many sources of interactions, there are still more factors that this study did not take into account, such as alcohol abuse, smoking, an impaired function of liver or kidneys which might require dose adaptions, risks from hypoalbuminia, a disregard of contraindications or a general overdose of pharmaceuticals. If these values were also taken into account, as should be the case in further studies, the number of patients at risk for interactions would be even higher. Our results might not be sufficient for generalizing since the study was only carried out at one hospital. The use of a distinctive drug interaction checking software presumably has a major influence on the results obtained. However, tools other than the one we finally decided to use did not find additional interactions in preliminary tests with random samples. Other limitations might be the lack of data especially regarding many CAM supplements and the unknown rate of a clinical manifestation of drug interactions found in our study but the example of QT elongation proves the relevance of this topic.

## Conclusion

The polypharmaceutical treatment and the high rate of patients at risk of drug–drug interactions require much more attention of physicians. Physicians should know about potential interactions in drugs they prescribe and should use a tool to search of potential interactions. A mandatory drug interaction check when prescribing a new drug could be realized by listing all patients’ medications in an electronic system functioning as an interaction checking tool as well. Also, a systematic integration of a pharmacist could be a model to reduce the risk of interactions. Patients should be asked about their use of CAM supplements. They must be enlightened by physicians and pharmacists about potential interaction risks regarding all types of interactions, including interactions with CAM substances or food as well as about time intervals to be complied with between food and drug intake. If there is no recommendation on taking a certain drug together with a meal, drugs should be taken separately and by using nonmineralized water to minimize the risk for food–drug interactions. Multicenter studies including more patients are required to capture precisely the prevalence and clinical relevance of different types of interactions. Our study provides the basis for this, as it identifies potential interactions. In cancer treatment interactions affecting conventional medications seem to be partly neglected and are an underestimated risk for the patients.

## Data Availability

Not applicable.

## Data availability

The datasets generated and analysed during the current study are available from the corresponding author on reasonable request.
